# Readiness to change among justice-involved young adults in an alternative sentencing program who screened positive for alcohol or drug risk

**DOI:** 10.1016/j.abrep.2022.100456

**Published:** 2022-09-17

**Authors:** Megan A. O'Grady, Susan Tross, Alwyn Cohall, Patrick Wilson, Renee Cohall, Stephanie Campos, Sin Lee, Curtis Dolezal, Katherine S. Elkington

**Affiliations:** a Department of Public Health Sciences, University of Connecticut, School of Medicine, USA; b Department of Psychiatry, Columbia University and the New York State Psychiatric Institute, USA; c Mailman School of Public Health, Columbia University, USA; d New York Presbyterian Hospital, USA; e HIV Center of Clinical and Behavioral Studies, USA

**Keywords:** Readiness to change, Young adults, Emerging adults, Criminal justice, Drug use, Alcohol use, Justice-involved, Motivation to change, Substance Use

## Abstract

**Introduction:**

Readiness to change is a key component of substance use behavioral change; yet little is known about readiness to change among justice-involved young adults. This study 1) describes readiness to change alcohol and drug use and 2) examines predictors of readiness to change alcohol and drug use among justice-involved young adults.

**Method:**

Justice-involved young adults (18–24 years; n = 137) who were positive on a validated alcohol and/or drug screening tool completed an interview assessing substance use, readiness to change, and reasons to quit. A multivariable linear regression model examined whether reasons to change and substance use severity, and interactions between these, predicted readiness.

**Results:**

More than half of participants were contemplating or had decided to quit/cut down substance use. Personal reasons to quit were positively related to readiness to change; interpersonal reasons were negatively associated.

**Conclusions:**

This study contributes information needed to design motivational interventions for substance use among justice-involved young adults. Personal reasons to quit using drugs are a potential intervention target.

## Highlights

• Justice-involved young adults are in need of tailored substance use interventions.
• Readiness to change is a key component of substance use behavioral change.
• Many justice-involved young adults were ready to change their substance use.
• Personal reasons to quit using drugs are a potential intervention target.

## 1 Background

Young adults, typically defined as those 18–24 years old, are overrepresented in the criminal justice system compared to the general population and have the highest illicit drug use prevalence of any age group (Siringil Perker & Chester, 2021). There is substantial prevalence of substance use disorder among frequently incarcerated justice-involved young adults (JIYA) (Chan et al., 2020). Few interventions target JIYA and there is a lack of research that can provide information on how to tailor interventions to them (Elkington et al., 2020, McMurran, 2009, Pederson et al., 2021, Prendergast et al., 2017, Tolou-Shams et al., 2021).

The Transtheoretical Model and previous research recognizes readiness to change, the degree to which an individual is motivated to change problematic behavior patterns, an essential component of behavioral change (Anderson et al., 2015, Austin et al., 2010, Knight et al., 2018, Pederson et al., 2021, Prochaska et al., 1992). Readiness is predictive of substance use outcomes among adolescents, young adults, and incarcerated youth (Alley et al., 2014, Austin et al., 2010, Clair et al., 2011, Hogue et al., 2010, Myers et al., 2016) and is a key target for brief motivational interventions. Studies among adults find that readiness to change may vary by criminal justice status and substance use treatment modality and point to the importance of readiness in predicting substance use outcomes (De Leon et al., 2000, Melnick et al., 2001, Melnick et al., 2014). While studies of readiness to change substance use have been conducted among young adults (Alley et al., 2018, Caviness et al., 2013, Goodman et al., 2015, Palfai et al., 2016), none that we are aware have been conducted among JIYA despite their higher risk profiles.

Several factors may be related to readiness to change, including reasons for wanting to quit substance use. Reasons to engage in behavior can be intrinsic, indicating desire to achieve internal rewards, or extrinsic, indicating desire to receive external rewards (Ryan & Deci, 2000). Intrinsic motives are reflected in personal reasons to quit (e.g., will like yourself better if you quit) and extrinsic motives in interpersonal reasons to quit (e.g., people you are close to will be upset if you don’t quit) (McBride et al., 1994, Smith et al., 2010). Research among young people in addiction treatment suggests that intrinsic reasons to quit are related to reductions in substance use, while extrinsic reasons predict less abstinence or are unrelated to substance use (Downey et al., 2001, Smith et al., 2010). Further, young adults have less interpersonal reasons to quit using substances than adolescents (Smith et al., 2010) and different reasons to quit than adults and adolescents (Copersino et al., 2006). As far as we are aware, studies have not examined whether reasons to quit predict readiness to change among young people who are not in addiction treatment. Young people in addiction treatment may differ from those not in treatment in terms of substance use severity and readiness or reasons to change. Therefore, there is great benefit to studying those in non-treatment settings who may have different substance use patterns and needs.

Another important factor in readiness to change may be substance use severity, but findings have been mixed. In a study of adolescents and adults seeking treatment, substance problems were the strongest treatment readiness predictor (Handelsman et al., 2005); studies among young people show substance use severity is related to greater readiness to change (Alley et al., 2014, Austin et al., 2010). Though, studies among adolescents have found a negative or no relationship between severity and readiness (Barnett et al., 2002, Battjes et al., 2003). It is possible that the relationship between readiness to change and reasons to quit may be moderated by substance use severity (Downey et al., 2001); however, this proposition has yet to be tested. For example, at higher levels of severity, individuals may have already faced severe negative external consequences (e.g., lost relationships or jobs), making external motivation less salient and internal motivation more salient.

The purpose of this article is to enhance understanding of readiness to change substance use among non-treatment involved JIYA who have screened positive for substance use risk by 1) describing levels of readiness to change alcohol and drug use and 2) examining the relationship between reasons for quitting, substance use severity, and readiness to change. Based on previous research on readiness and substance use outcomes (Smith et al., 2010), we hypothesized that more personal reasons for quitting, but not interpersonal reasons, would predict higher readiness to change. We also explored whether reasons for change moderated the relationship between substance use severity and readiness to change (i.e., relationship would be strongest among those with more personal reasons for quitting); a relationship previously proposed and potentially important for informing intervention development (Downey et al., 2001), but untested to date. Findings may inform adaptation of motivational interventions for JIYA.

## 2 Materials and methods

### 2.1 Setting, participants, and procedures

Data were drawn from the baseline assessment of a randomized controlled trial testing an intervention to promote substance use and STD/HIV service readiness and risk reduction (Elkington et al., 2020). The study was conducted at an alternative sentencing program (ASP) in a criminal courthouse in the Brooklyn borough of New York City. JIYA were eligible to participate if they were 18–24 years old, enrolled in the ASP, conversant in English, and reported engaging in past year unprotected vaginal or anal intercourse. JIYA were informed of the study by either ASP or study staff. If interested, study staff conducted a brief eligibility screen. If eligible, a baseline interview was scheduled and informed consent obtained by a research assistant. This study has been approved by the New York State Psychiatric Institute Institutional Review Board (protocol #7574). For this paper, only participants randomized to the intervention group in the parent study were included because, by design, intervention participants, not controls, were administered the substance use screenings that classified risk. Further, we only included those who screened positive on the substance use screening tools (see Materials) because we were interested in readiness to change among those with some level of risk (e.g., those who would be eligible for a brief intervention in standard practice)(Babor et al., 2001, Roy-Byrne et al., 2014).

### 2.2 Materials

#### 2.2.1 Demographics, substance use, and severity

Descriptive substance use information was collected using the AIDS-Risk Behavior Assessment (ARBA) (Donenberg et al., 2001). Severity of drug use was assessed using the Drug Abuse Screening Test (DAST-10), a 10-item, yes/no screener with scores ranging from 0 to 10 (Skinner, 1982, Yudko et al., 2007) and scores of ≥ 1 considered positive. Severity of alcohol use was collected using the Alcohol Use Disorders Identification Test (AUDIT) (Babor et al., 2001), a 10-item (coded from 0 to 4) screening tool with scores that can range from 0 to 40, and scores ≥ 8 considered positive. For descriptive purposes, we collected information on incarceration history, demographics, socio-economic status, and mental health (Brief Symptom Inventory-18) (Derogatis, 2001).

#### 2.2.2 Reasons for quitting

The Reasons for Quitting Scale, from the substance use section of the Global Appraisal of Individual Needs-Initial (GAIN-I) Version 5.0 (Dennis, 2008), contains 33 yes/no items assessing reasons to quit using drugs or alcohol. Originally developed for adults, it was adapted for use with adolescents; previous validations found a two-factor solution measuring personal and other health consequences (Personal; 20 items; e.g., Will be able to think more clearly) and pressure from family and friends (Interpersonal; 13 items; e.g., Close ones will stop nagging you if you quit)(McBride et al., 1994, Smith et al., 2010). We used these subscales for analysis; scores are a count of items endorsed with a yes. Previous studies show good reliability, predictive validity, and positive correlations with other motivation scales, drug use outcomes, and treatment completion (Smith et al., 2010). The scale is also clinically relevant, having been used to provide individualized feedback in brief motivational substance use interventions (Sample, 2001).

#### 2.2.3 Readiness to change alcohol and drug use behavior

A validated visual analog of a contemplation ladder assessed readiness to change (Hogue et al., 2010); two separate ladders (Appendix A), one for drug and one for alcohol, were used. The ladder has rungs with seven associated anchor statements, yielding scores from 1 to 7. Participants could select a rung between two anchor statements (e.g., 2.5). The ladder shows good validity and is predictive of substance use and participation in treatment (Hogue et al., 2010). Such ladders do not categorize individuals into discrete stages of change, but instead provide a single continuous metric of readiness to change behavior (Hogue et al., 2010).

### 2.3 Analysis

Descriptive statistics were calculated for all variables. Two multivariable linear regression models were fit: 1) to examine whether personal and interpersonal reasons to quit and drug use severity predicted scores on the drug contemplation ladder and 2) to test interaction terms between reasons to quit and severity. Three control variables were included: sex, ethnicity (Latino/Hispanic vs not), and race (Black/African-American vs not). There was not a large enough alcohol risk sample to conduct a similar analysis with the alcohol ladder.

## 3 Results

### 3.1 Description of demographics, substance use, and reasons to quit

Table 1 provides demographics and descriptives. The sample was mainly Black and/or Latinx and male. Of the 137 participants, 135 screened positive on the DAST-10 and 42 screened positive on the AUDIT; two individuals screened positive on only the AUDIT, the other 40 also screened positive on the DAST-10. Over half of the sample fell into the intermediate or substantial/severe range on the DAST-10. Interestingly, few participants screened positive for alcohol risk; only 11 % were in the harmful/severe range on the AUDIT. Marijuana was the most frequently used drug (∼90 % in the past three months; Appendix A). Alcohol was also frequently used (81 %). Other notable substances used include opioids (16 %), benzodiazepines (12 %), and ecstasy (10 %).

**Table 1:** Descriptive Characteristics of Sample (n = 137).

Variable	M (SD) or N(%)
Age	20.68 (2.00)
Gender	
Male	101 (74 %)
Female	34 (25 %)
Transwoman	1 < 1 %
Genderqueer/Nonconforming	1 < 1 %
Race/Ethnicity*	
Non-Hispanic	
African American/Black	70 (52 %)
More than 1 race	9 (7 %)
Other	8 (6 %)
Hispanic	
African American/Black	24 (18 %)
More than 1 race	21 (15 %)
Other	4 (3 %)
Living Situation*	
Homeless	14 (10 %)
College housing	0 (0 %)
Living rent-free	71 (52 %)
Renting	41 (30 %)
Owns apartment/house	2 (2 %)
Other	8 (6 %)
Ever Homeless	57 (42 %)
Education	
Less than HS grad	66 (49 %)
HS grad/GED	65 (48 %)
College grad	5 (4 %)
Currently employed	43 (31 %)
Currently married or living with a partner	19 (14 %)
Has child/children	27 (20 %)
Criminal Justice History	
Any arrest, past year	107 (78 %)
Night in Jail/Prison, past year	32 (23 %)
# Times Arrested, past year Range	1.99 (2.44) 0–15
# Juvenile justice contacts, lifetime Range	1.86 (4.30) 0–25
# Adult justice contacts, lifetime Range	7.19 (13.72) 0–97
Psychological Distress (BSI-18 T scores)†	
Somatization	54.23 (10.30)
Depression	54.91 (10.77)
Anxiety	53.02 (11.25)
Global Severity Index	55.28 (11.23)
DAST-10 Score	3.34 (2.19)
None (0)	2 (1 %)
Low (1–2)	61 (45 %)
Intermediate (3–5)	50 (36 %)
Substantial/Severe (6 + )	24 (18 %)
AUDIT Score	6.20 (6.55)
No/Low risk (<8)	95 (69 %)
Hazardous (8–15)	27 (20 %)
Harmful (16–19)	6 (4 %)
Severe (20 + )	9 (7 %)
Reasons to quit: Personal subscale (0–20)	10.03 (6.29)
Reasons to quit: Interpersonal (0–13)	3.73 (2.91)
Drug Ladder Score (1–7)	3.29 (2.08)
Alcohol Ladder Score (1–7)	3.50 (2.27)

* Ns do not sum to 137 due to 1 missing data point; percentages are of those with non-missing data. ^†^T scores ≥ 63 indicate psychological distress on the BSI-18.

The top two endorsed reasons to quit were “to show yourself that you can quit if you really want to” (76 %) and “because you want to do better in life” (74 %). Surprisingly, given the sample’s criminal justice involvement, drug testing and legal problems were not highly endorsed (19 % and 16 % respectively).

### 3.2 Readiness to change drug and alcohol use behavior

The average scores (Table 1) for the drug and alcohol ladders indicated that participants were thinking about cutting down their drinking or drug use, but not about quitting all together. The ladder score distributions suggest that 56 % and 60 % of participants expressed readiness to reduce or quit their drug or alcohol use, respectively (full score distributions available in Appendix A).

### 3.3 Predictors of readiness to change drug use behavior

Both regression models (Table 2) were statistically significant (Model 1: R^2^ = 0.22, *F* (6,126) = 5.86, *p* =.000; Model 2: R^2^ = 0.24, *F* (8,124) = 4.97, *p* =.000). In model 1, there was a significant positive relationship between personal reasons to quit and the ladder score (i.e., more personal reasons related to higher readiness to change). There was a significant negative relationship between interpersonal reasons to quit and readiness to change. Drug use severity (i.e., DAST-10) was not significantly related to readiness to change. In model 2, the interaction terms between reasons to change and severity were not statistically significant.

**Table 2:** Multiple Regression: Reasons to Quit and Drug Use Severity as Predictors of Drug Ladder (i.e., readiness to change) Scores (n = 135).

Variable	B (SE) 95 % CI	β	*t score*	*p value*
Model 1				
Reasons to quit: Personal	0.17 (0.03) 0.10–0.24	0.51	4.94	0.000*
Reasons to quit: Interpersonal	−0.15 (0.07) −0.29 - −0.01	−0.21	−2.09	0.039*
DAST-10 score	0.04 (0.09) −0.13–0.21	0.04	0.48	0.630
Model 2				
Reasons to quit: Personal	0.27 (0.07) 0.14–0.39	0.80	4.10	0.000*
Reasons to quit: Interpersonal	−0.42 (0.15) −0.72 - −0.11	−0.58	−2.70	0.008*
DAST-10 score	0.07 (0.18) −0.28–0.43	0.08	0.42	0.676
Personal reasons X DAST-10	−0.03 (0.02) −0.06–0.00	−0.52	−1.69	0.094
Interpersonal reasons X DAST-10	0.08 (0.04) 0.00–0.15	0.55	1.93	0.057

Note.

* = significant at *p* <.05, Higher scores on drug ladder = more readiness to quit/cut down. Adjusted for Hispanic/Latino (yes/no); sex (Male/female); Race (black vs other).

## 4 Discussion

JIYA have an elevated burden of substance misuse, yet there is little understanding of their readiness to quit or cut down. Results of this study showed that among JIYA who screened positive for substance use risk, most risk was related to drug, rather than alcohol use, and more than half were contemplating or had decided to quit or cut down drug or alcohol use. This is significant given that the sample was not in or seeking treatment and legal consequences nor drug testing were strongly endorsed as reasons to quit substance use, which have been identified as important extrinsic motivators in other studies (Gregoire & Burke, 2004). Findings suggest JIYA may be receptive to interventions targeting readiness to change substance use.

Our hypothesis that more personal reasons to quit would predict higher readiness to change was confirmed. This is consistent with previous research and adds to the literature by showing intrinsic reasons are positively related to readiness to change—an outcome not previously examined. Additionally, we found a negative relationship between number of interpersonal reasons to quit and readiness to change. While we did not predict this, a previous study (Downey et al., 2001) found extrinsic motivation was negatively associated with abstinence among people in addiction treatment. The smoking literature also shows successful smoking abstinence related to lower extrinsic and higher intrinsic motivation (Curry et al., 1997). We did not find a significant relationship between drug use severity and readiness to change. Mixed findings in previous research on this relationship lead to questions about whether severity instead moderates the relationship between reasons to change and readiness (Downey et al., 2001); therefore, we explored this possibility but did not find this was the case. More research is needed to understand the mixed relationship between severity and readiness.

The study sample was mostly male, Black, and Latinx, mirroring the national justice population (Vaughn et al., 2018). This is an important sample from a public health perspective, whose needs, challenges, and strengths deserve focused attention. Latinx and Black individuals with substance use problems have greater unmet treatment needs, lower treatment retention, and more severe substance use consequences (Austin et al., 2010). Readiness to change among young people has largely been studied among White non-Hispanic populations (Austin et al., 2010), and almost nothing is known about reasons to quit and readiness to change substance use among JIYA. Young adults are frequently not recognized as a developmentally unique group within justice systems (Bory et al., 2021, Siringil Perker and Chester, 2021). Continued substance use among JIYA can further deepen involvement in the justice system and contribute to cyclic justice engagement (Chan et al., 2020) that becomes a barrier to meeting key developmental young adulthood milestones (e.g., education completion, long-term relationships) (Arnett, 2000, Arnett, 2005).

This study had limitations, including small sample size. This could have affected our ability to detect a significant interaction between the DAST-10 and reasons to quit and may warrant future investigations to confirm. While study staff took considerable effort to ensure participants did not feel coerced to participate and that responses would be confidential, there is a possibility of social desirability bias. Data was from a larger study which recruited individuals based on past year sexual risk behavior, therefore may not be representative of all JIYA.

Despite limitations, findings fill a critical knowledge gap and have implications for the design and implementation of motivational interventions for Black and Latinx JIYA. For example, personal reasons to quit are a potential target during motivational interventions. Given the large gap in services for JIYA, new models are needed partnering behavioral health with justice agencies (DeLucca et al., 2022). This study was conducted with ASP participants in a courthouse suggesting this environmental and organizational context, considered important for JIYA behavioral health service implementation (Bowser et al., 2019), is potentially favorable for substance use services implementation—future research should examine this potential.

Data and analysis code are available by reasonable request to the last author. The design of this study has been pre-registered on ClinicalTrials.gov (NCT03369249).

## CRediT authorship contribution statement

**Megan A. O'Grady:** Conceptualization, Writing – original draft, Writing – review & editing, Funding acquisition. **Susan Tross:** Conceptualization, Writing – original draft, Conceptualization, Writing – review & editing, Funding acquisition. **Alwyn Cohall:** Conceptualization, Funding acquisition. **Patrick Wilson:** Conceptualization, Writing – review & editing, Funding acquisition. **Renee Cohall:** Conceptualization, Writing – review & editing, Funding acquisition. **Stephanie Campos:** Writing – review & editing, Project administration. **Sin Lee:** Data curation, Writing – review & editing, Investigation. **Curtis Dolezal:** Formal analysis, Methodology, Data curation. **Katherine S. Elkington:** Conceptualization, Writing – original draft, Writing – review & editing, Project administration, Funding acquisition.

## Declaration of Competing Interest

The authors declare that they have no known competing financial interests or personal relationships that could have appeared to influence the work reported in this paper.

## Figures and Tables

**Table:** Participant substance use patterns (N = 137)

Substance^1^	Ever Used	Used Past 3 Months	Frequency of Use Past 3 Months^2^	
	N (%)^3^	N(%)^3^	Median	Range
Alcohol	131 (96 %)	108 (81 %)	6	1–420
Marijuana	133 (97 %)	120 (90 %)	100	1–1350
Cocaine	25 (18 %)	10 (7 %)	3	1–40
Prescription Stimulants	20 (15 %)	6 (4 %)	2	1–10
Opiates/Narcotics	44 (32 %)	22 (16 %)	3	1–900
Benzodiazepines	44 (33 %)	16 (12 %)	7	1–180
Psychedelics	27 (20 %)	11 (8 %)	2	1–43
Synthetic Marijuana	18 (13 %)	0 (0 %)	NA	NA
Ecstasy	38 (28 %)	14 (10 %)	3	1–24

**Table:** Distribution of Drug and Alcohol Ladder Scores.

Ladder Score	Ladder Label	Drug Use % (N = 135)	Alcohol Use % (N = 42)
7	I have decided to quit using drugs/drinking alcohol and plan never to use drugs/drink again	9 %	0 %
6–6.5	I have decided to quit using drugs/drinking alcohol, at least for now	9 %	17 %
5–5.5	I am close to making a decision to quit using drugs/drinking alcohol	10 %	10 %
4–4.5	I am thinking about quitting using drugs/drinking alcohol altogether, but I still have not made definite plans	17 %	19 %
3–3.5	I am thinking about cutting down on my drug use/drinking, but I am not thinking about quitting drugs/drinking altogether	11 %	14 %
2–2.5	I might have a problem with drugs/drinking, but I do not intend to cut down or quit now	12 %	9 %
1–1.5	I do not have a problem with drugs/drinking, and I do not intend to cut down or quit now	32 %	31 %

## Appendix A Participant substance use patterns and readiness to change

*Note*. ^1^ Substances not shown because used by<10 % of participants (ever) include crack, methamphetamine, heroin, methadone, barbiturates, and inhalants. ^2^ Times used among those who used the substance in the past 3 months. ^3^ Some variables have missing data; % are of those with non-missing data.

Note: Ns reflect participants who screened positive on the DAST (N = 135) and AUDIT (N = 42).
